# Medical errors – I : The problem

**DOI:** 10.4103/0019-5545.64580

**Published:** 2010

**Authors:** G. Swaminath, R. Raguram

**Affiliations:** Department of Psychiatry, Dr B R Ambedkar Medical College, Kadugondanahalli, Bangalore- 560 045, Karnataka, India; 1 Department of Psychiatry, Kempegowda Institute of Medical Sciences, Bangalore, Karnataka, India

## FIRST, DO NO HARM

‘*I felt a sense of shame like a burning ulcer. This was not guilt: guilt is what you feel when you have done something wrong. What I felt was shame: I was what was wrong. And yet I also knew that a surgeon can take such feelings too far. It is one thing to be aware of one’s limitations. It’s another to be plagued by self-doubt.’[1]*

Atul Gawande aptly describes the angst experienced by a doctor on discovering that the increased suffering in a patient was due to a slip-up on the part of the treating team. Medical errors can have a disastrous effect on patients, staff, and institutions. Medical errors are too often a taboo subject. They haunt the conscience of those involved and the medical personnel naturally find them difficult to discuss.[2]

As a rule, hospitals and healthcare professionals profess and aim to provide the safest care possible. However, things may go wrong, harm can be done, and patients can inadvertently be hurt as a consequence of medical care.[3] Wu *et al*.[4] define an ‘Error’ as a commission or an omission, with potentially negative consequences for the patient, which would have been judged wrongly by skilled and knowledgeable peers at the time it occurred, independent of whether there were any negative consequences. Adverse events or iatrogenic injury, as a consequence of an error results in prolongation of hospital stay, morbidity or disability at discharge, or death.[5] As the definition is rather stringent, it is possible that there is an underestimation of adverse events, as many errors do not result in injury because they are caught in time, the patient is resilient or because of plain good luck.[3] These adverse events must be distinguished from unfavorable side effects, which are unpredictable or unavoidable complications that may occur during the appropriate application of the best practice, whereas, the former result from an error.

## TO ERR IS HUMAN

*To Err Is Human*,[6] the report from the National Academy of Sciences, Institute of Medicine (2000), estimated that as many as 98,000 people die every year in the US because of mistakes committed by medical professionals in hospitals. According to the report, more people die annually from medical errors than from motor vehicle accidents, breast cancer or AIDS — the three causes that receive far more public attention.[7] In the Harvard Medical Practice Study,[8] a review of 31,429 records of patients identified an adverse event in 3.7% of the admitted patients; 43% of these adverse events caused at least moderate impairments to the patients. In 69% of these cases, the adverse events were considered preventable (implying error), as opposed to events considered to be non-preventable, that is, anticipated, but with unavoidable complications. Another study using the same method of reviewing medical records[9] found that an adverse event occurred in 16.6% of 14,179 admissions in hospitals in New South Wales and South Australia, resulting in permanent disability in 13.7% of the affected patients and death in 4.9%. This study found that 51% of the cases were due to error. Unfortunately, similar studies are lacking in psychiatric populations, with few reports published on rates of error or adverse events, and there has been little commentary on related methodological issues.[10] Some prominent psychiatric journals do not even include ‘medication errors’ or ‘adverse drug events’ as keyword options for the submitted manuscripts.[10]

Among adverse event surgery forms the source in almost half of the cases, followed by complications resulting from medication treatment, therapeutic mishaps, and diagnostic errors.[3] Diagnostic errors were the most frequent non-operative errors. However, most research has focused on medication errors rather than diagnostic errors. Among the therapeutic mishaps and diagnostic errors, those of omission outnumbered those of commission, two-fold.[3] Errors of omission were failure of action,[3] such as: (i) missed diagnosis, for example, missing depressive syndrome in psychotic patients or in those with predominant somatic symptoms, and consequently not striving to treat it; missing a comorbid medical illness, thereby slowing the improvement of the psychiatric illness; (ii) Omission or delayed evaluation of important parameters, for example, to delay / omit the evaluation of blood counts following administration of clozapine; or to delay / omit periodic estimation of serum lithium: or (iii) failure to prescribe the needed drug treatment, for example, failure to correct and replenish daily fluid and / or calories (after the treating team and relatives heave a sigh of relief) after sedating a violent patient; or failure to administer Vitamin B1 prior to administering dextrose fluids in alcoholics. Errors of commission are incorrect actions, such as, administering the wrong drug to the patient, often at the wrong time,[3] choosing carbamazepine or valproate for a pregnant woman, use of lithium in renal disorder or prescribing antipsychotics, or misconstruing obsessive thoughts or imagery for delusions or hallucinations, respectively.

Among therapeutic mishaps, the rare operation or removal of a wrong limb is appropriately perceived as a grave offense, as there is an immediate individual tragedy involved. However, the more common failure to detect a depressive syndrome in a patient by the physician, resulting in suicide, or the failure to start a β – blocker following admission for myocardial infarction^11^ is lost in population statistics. There are many examples of these apparent paradoxes, such as the public mobilization to help in the individual tragedy of a little girl who falls in a well versus our inattention to the population tragedy of AIDS in sub-Saharan Africa.[11]

## THE THREE ‘A’S: HOW AND WHY OF MEDICAL ERRORS

Errors often occur not because physicians have inadequate knowledge or are incompetent, but that they have a tendency to get stuck in a particular mode of thinking.[12] These ‘cognitive errors’ such as incorrect diagnosis or choosing the wrong medication are more likely to have been preventable and more likely to result in permanent disability than technical errors.[13] These cognitive pitfalls are part of human thinking, biases that cloud logic when we make judgments under conditions of uncertainty and time pressure.[14] These cognitive errors reportedly give rise to three principal biases: (i) ‘anchoring,’ where a person overvalues the first data he encounters and so is skewed in his thinking; (ii) ‘availability,’ where recent or dramatic cases quickly come to mind and color the judgment about the situation at hand; and (iii)’attribution,’ where stereotypes can prejudice thinking, therefore, conclusions arise not from data but from such preconceptions.[14]

In one important study, medication errors caused 20% of all injuries, with 18% of these considered to be preventable.[3] As for prescribing errors in psychiatry, almost 60% of them were ‘clinical’ in nature.[15] They originated from a lack of understanding of what was being prescribed, what the correct dose should be, how the drug worked, and the drug interactions that might be anticipated.[15] Monitoring errors were also common, particularly prescribing clozapine in the absence of satisfactory blood results.[15] However, among errors across all specialties, 56% occurred during drug ordering (prescribing a wrong drug or incorrect dosage) and 24% during drug administration, of which non-availability of the drug, omission to give the drug when available, and giving the wrong dose were the types of errors that predominated.[3]

At an empirical level then, errors can be[16]

- Slips — On decision after weighing the pros and cons in choosing between SSRIs, tag the chosen SSRI with the dosage of another, for example, Escitalopram 50 mg, or prescribing a wrong brand among similar sounding generic or brand names, for example, atomoxetine / tomoxifen or Serenace (haloperidol) / Serenata (sertraline) / Sarotena (amitriptyline). India unfortunately has brands with confusing nomenclature adding to the problem.[17]
- Lapses — On deciding to change to a sustained release / long-acting preparation from an short-acting one and forgetting to strike off the immediate action drug in the treatment chart so the patient gets both, that is, both sodium valproate and divalproex prescribed in the treatment chart.
- Mistakes — wrong choice of drug, not considering comorbid states, for example, olanzapine in comorbid diabetic state, propranolol in an asthmatic state.

Many consumers of mental health services suffer needlessly as a result of being given the wrong medication or the wrong dosage of the right medication or from other mistakes involving medication. There are seven common medication mistakes: prescribing incorrectly as a result of misdiagnosis, excessive dosages of medications, too many drugs, downplaying side effects, overlooking the consumer’s expertise, discouraging consumers from learning about their medications, and the prescription-sheet relationship between the psychiatrist and consumer.[18] Prescription writing errors (77.4%) were most common in one systematic analysis, while decision-making errors accounted for 22.6% of the errors.[19] In 53.5% of the cases the prescribed drug had been administered before the error was detected. Most errors were of doubtful or minor importance, but 4.3% were deemed likely to result in serious adverse effects or death.[19]

The recent Equip Study[20] (Errors — Questioning Undergraduate Impact on Prescribing) commissioned by the UK General Medical Council (GMC), investigated prescribing errors by foundation trainees (doctors in the first two years after leaving medical school) and compared them with errors made by more senior doctors. The error rate was 8.4 and 10.3% for doctors in the first two years, while it was 5.9% for consultants; 1.7% of the errors were potentially lethal. What is significant about the error rates released by the GMC is that almost one in ten hospital prescriptions were wrong, with the error rate for consultants being as high as one in 20, while that the most junior doctors were no worse than the average. It is important to note that very little is known about the incidence, pattern, and causes of errors in mental health care in private practice settings, as most of the studies have been done in hospital settings.[21]

The recommendations of the Equip study authors includes introduction of a standard drug chart throughout the National Health Service, which would reduce errors as well as build a ‘safety culture’ in clinical practice. They also recommend that practical prescribing components and practice be included in medical education and CME programs for undergraduates, postgraduates, and higher specialists.

## HARM REDUCTION

Reduction of errors has become an important marker of the quality of care and is included in the clinical performance indicators. By identifying and studying adverse events, we can learn lessons and change practice in a manner that will make such events less likely in the future, and hence improve the safety of patients and the quality of care.[3] Errors indicate a breakdown in the system or wrong decision-making.[3] In an important book ‘How Doctors Think’, Dr Jerome Groopman has argued that errors in thinking rather than errors in technique, contribute to mistakes.[22] However, Groopman omits examination of errors in psychiatric practice. This lacuna has been addressed in a recent article ‘How Psychiatrists Think’ where Crumlish and Kelly outline a range of ten cognitive errors that occur commonly in psychiatric practice and identify confirmation bias (tendency to seek out information which supports one’s beliefs, and ignore contradictory information) to be the most common one.[23]

Errors cannot be ignored. They must be recognized, their causes analyzed, and preventive measures taken.[3] We should try to understand the causes of errors, to install an informative reporting system of adverse events as an essential prerequisite, to measure them, and to choose the best approaches for minimizing the harm to patients.[3] Patient safety is to be improved by the collective effort of all those involved in mental health care.

## References

[1] Gawande A (2002). Complications.

[2] Penson RT, Svendsen SS, Chabner BA, Lynch TJ, Levinson W (2001). Medical mistakes: a workshop on personal perspectives. Oncologist.

[3] Eldar R (2002). Understanding and preventing adverse events. Croat Med J.

[4] Wu AW, Cavanaugh TA, McPhee SJ, Lo B, Micco GP (1997). To tell the truth: ethical and practical issues in disclosing medical mistakes to patients. J Gen Intern Med.

[5] Brennan TA, Localio RJ, Laird NL (1989). Reliability and validity of judgments concerning adverse events suffered by hospitalized patients. Med Care.

[6] Kohn LT, Corrigan JM, Donaldson MS, editors (2000). To err is human: building a safer health system.

[7] Kalantri SP (2003). Medical errors and ethics. Indian J Anaesth.

[8] Brennan TA, Leape LL, Laird NM, Hebert L, Localio AR, Lawthers AG (1991). Incidence of adverse events and negligence in hospitalized patients: results of the Harvard Medical Practice Study I. N Engl J Med.

[9] Wilson RM, Runciman WB, Gibberd RW, Harrison BT, Newby L, Hamilton JD (1995). The Quality in Australian Health Care Study. Med J Aust.

[10] Grasso BC, Bates DW (2003). Medication errors in psychiatry: are patients being harmed?. Psychiatr Serv.

[11] Hofer TP, Kerr EA, Hayward RA (2000). What is an error?. Eff Clin Pract.

[12] Price M (2010). The antidote to medical errors. Monitor Staff.

[13] Wilson RM, Harrison BT, Gibberd RW, Hamilton JD (1999). An analysis of the causes of adverse events from the Quality in Australian Health Care Study. Med J Aust.

[14] Groopman JE (November 5 2009). Diagnosis: What doctors are missing. The New York Review of Books.

[15] Paton C, Benham SG (2003). Prescribing errors in psychiatry. Psychiatr Bull.

[16] Dean B, Schachter M, Vincent C, Barber N (2002). Causes of prescribing errors in hospital inpatients: a prospective study. Lancet.

[17] Shamasundar C (1992). Prescriber’s nightmare. Indian J Psychiatry.

[18] Blaska B (1990). The myriad medication mistakes in psychiatry: a consumer’s view. Hosp Community Psychiatry.

[19] Stubs J, Haw C (2006). Prescription errors in psychiatry: A multi center study. J Psychopharmacol.

[20] (2009). Editorial. How to reduce prescribing errors. Lancet.

[21] Maidment ID, Lelliott P, Paton C (2006). Medication errors in mental health care: a systematic review. Qual Saf Health Care.

[22] Groopman J (2008). How doctors think.

[23] Crumlish N, Kelly BD (2008). How Psychiatrists Think. Adv Psychiatr Treat.

